# Optimizing Reduction Guide Stability in Osteotomy Using Patient-Specific Instrumentation: A Basic Guideline

**DOI:** 10.1177/23259671241275124

**Published:** 2024-12-10

**Authors:** Michel Meisterhans, Christoph Zindel, Bastian Sigrist, Sandro F. Fucentese, Lazaros Vlachopoulos

**Affiliations:** † Department of Orthopaedics, Balgrist University Hospital, University of Zurich, Zurich, Switzerland; ‡ Research in Orthopedic Computer Science (ROCS), Balgrist University Hospital, University of Zurich, Zurich, Switzerland; Investigation performed at Department of Orthopaedics, Balgrist University Hospital, University of Zurich, Zurich, Switzerland

**Keywords:** PSI, knee, osteotomy, biomechanics of bone, biomechanics, osteotomy, reduction guide, stability, finite element analysis

## Abstract

**Background:**

The use of patient-specific instruments (PSIs) for osteotomies is becoming more popular in orthopaedic surgery for correcting mechanical axis and posttraumatic deformities. However, the PSI reduction guides have great potential for intraoperative deformation, which adversely affects the accuracy of the procedure.

**Purpose:**

To conduct a finite element analysis (FEA) to analyze different design parameters to improve the intraoperative stability of the reduction guides.

**Study Design:**

Descriptive laboratory study.

**Methods:**

A reduction guide with a rectangular cross section and four 4-mm K-wire slots was simplified, and the following parameters were modified: width, height, profile design, K-wire thickness, and positions. Bending and torsional moments were applied to the guide construct and guide deformation and equivalent stress were determined using FEA.

**Results:**

Increasing the profile height by 25% resulted in a 44% reduction in guide deformation for bending (37% for torsion). A 25% increase in profile width led to an 18% deformation reduction for bending (22% for torsion). Transverse K-wire slots resulted in 51% less deformation in torsion compared with longitudinally oriented slots. Placing the central K-wire slots 25% closer to the osteotomy reduced guide deformation by 20% for bending and 11% for torsion.

**Conclusion:**

The most effective methods to increase reduction guide stability are to increase the guide height and reduce the central K-wire distance to the osteotomy.

**Clinical Relevance:**

When performing opening or closing wedge osteotomies, which mainly involve bending of the guide, a high-profile guide and longitudinally oriented K-wire slots should be used. When torque is expected as in rotational osteotomies, the K-wire holes in guides should be oriented transversely to reduce intraoperative deformation.

Computer-assisted 3-dimensional (3D) planning and patient-specific instruments (PSIs) have gained increasing importance in orthopaedic surgery by providing precise and customized treatment options for patients with mechanical axis and post-traumatic deformities in their upper and lower extremities.^4,15,21,24^ The reduction can be performed either directly by the manipulation of the fragments or indirectly by the reduction with the implant. For the direct manipulation of the fragments, a K wire–based reduction guide or a fragment reduction guide can be designed.^13,16^ For the indirect reduction with the implant, a predrilled screw hole guide, a prebent plate, or a ramp guide can be used.^23,25^ The implant-independent reduction guide is widely used in PSI procedures due to its simple technique and free implant choice for the surgeon.^23,25,26^

A PSI osteotomy surgery typically consists of the following steps^25^: (1) positioning of the basic guide for registration of the preoperative plan to the intraoperative situation, (2) performing of the osteotomy through the basic guide with integrated cutting slot or an additional osteotomy guide, (3) reduction of the fragments with a K wire–based reduction guide (Figure 1), and (4) fixation of the fragments with a plate or screws.

**Figure 1.**
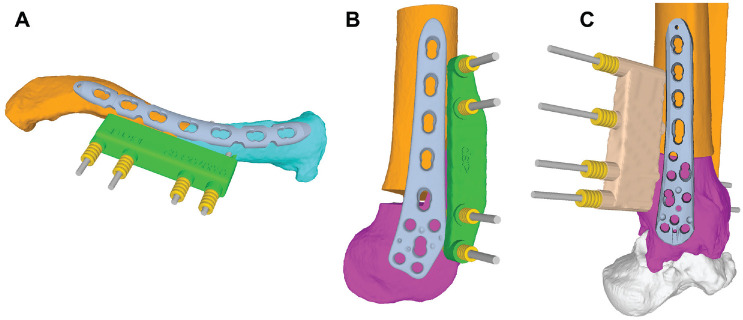
Examples of reduction guides for different indications. (A) Corrective osteotomy of a clavicular malunion. (B) Lateral opening wedge distal femoral osteotomy. (C) Corrective rotational osteotomy of the distal tibia.

PSIs have been widely recognized in reducing the surgeon’s learning curve and the need for intraoperative fluoroscopy, as well as in shortening the surgical duration.^10^ The use of PSI in high-tibial osteotomies (HTO) leads to accurate corrections.^8,12,18^ When compared to traditional surgical techniques, a meta-analysis published in 2022 that analyzed 10 studies focusing on PSI found that adopting PSI resulted in a nonsignificantly lower outlier rate and a nonsignificantly higher accuracy.^5^ The accuracy of the procedure depends on the preoperative 3D analysis, the planning and design of the PSI guides on the one hand, and on the stability of the reduction guide on the other hand. In case of excessive soft tissue tensioning or the remaining bone hinge during a osteotomy high-bending moments and torque is applied on the reduction guide and can lead to deformation of the guides.^3,11,22,25^ This then leads to an inaccuracy of the planned correction and is a possible reason why the PSI technique is not significantly superior to conventional osteotomy techniques in terms of accuracy. This problem is frequently observed intraoperatively but rarely reported in the literature.^11^

Therefore, there is a growing need to improve the stability of these guides to ensure that they remain accurate during surgery.^11^ To date, there is no study available investigating PSI reduction guide stability or optimizing its design to improve intraoperative stability. To address this issue, different design parameters that could improve the stability of reduction guides have been analyzed in this study using a finite element analysis (FEA).

## Methods

### Development of Reduction Guide Models

**Figure 2.**
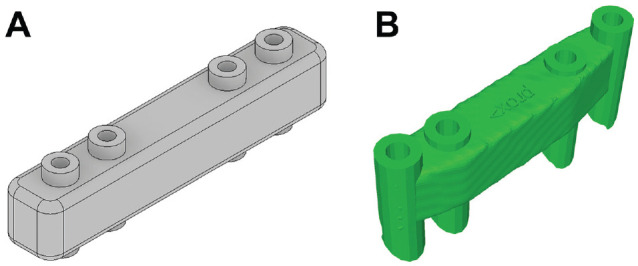
(A) Design of a simplified standard reduction guide used for the finite element analysis, corresponding to the G 00 basis design based on (B) the dimension of a reduction guide from lateral opening wedge distal femoral osteotomy to the right.

**Figure 3.**
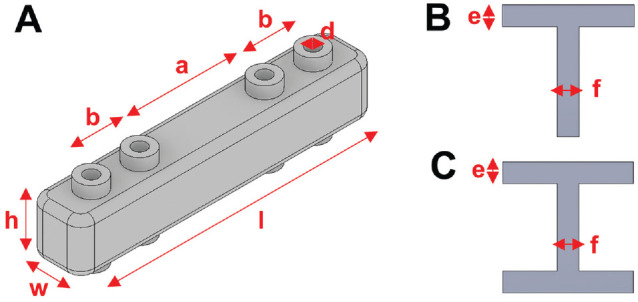
(A) Reduction guide with labeled dimensions, which are varied for the different guide configurations: length (*l*), width (*w*), height (*h*), intrafragmentary K-wire distance (*b*), interfragmentary K-wire distance (*a*), and K-wire slot diameter (*d*). Crosssection of a T-profile (B) and H-profile (C) and its dimensions beam height (e) and beam width (f).

A PSI reduction guide for a lateral opening wedge distal femoral osteotomy with 4 K-wire slots (2 per osteotomy side) was used as a reference (Figure 2, A and B). Based on this, a simplified reduction guide was designed using Autodesk Inventor (Autodesk Inc) with the length of 85 mm, width of 15 mm and height of 15 mm. The K-wire slots were designed to accommodate K-wires with a diameter of 4 mm. The interfragmentary K-wire distance (*a*) was 36 mm, whereas the intrafragmentary K-wire distance (*b*) was set to 14 mm (Figure 3).

### Factors of Interest

**Figure 4.**
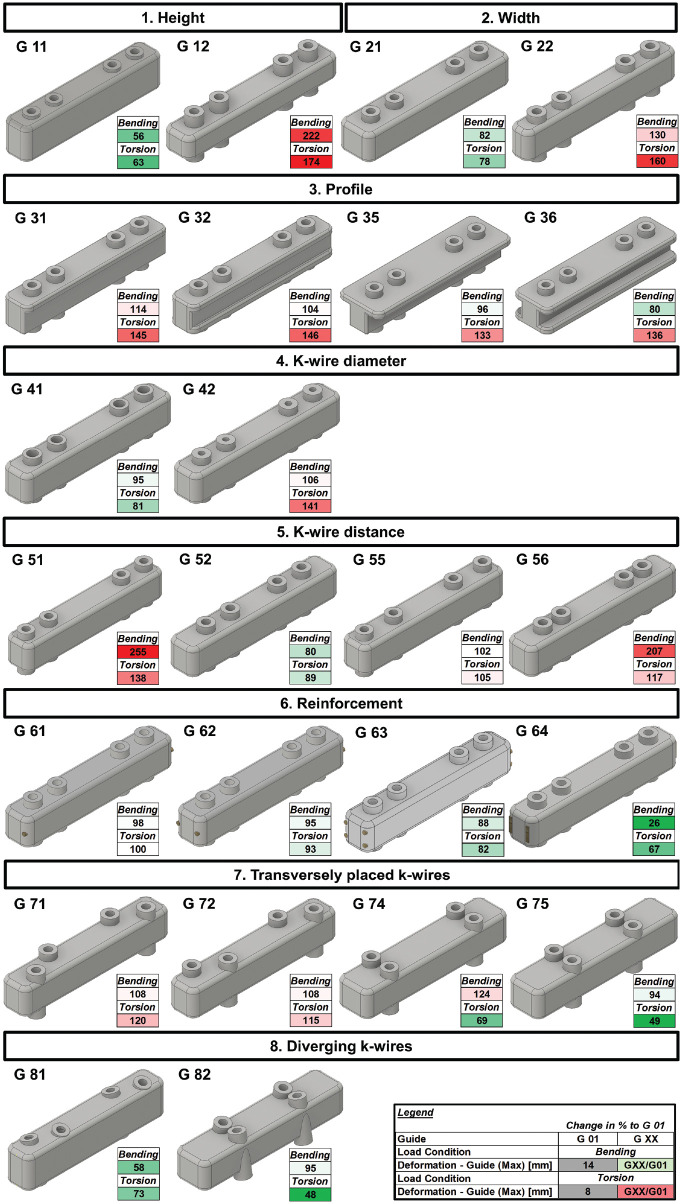
Overview of the different guide designs and guide deformation compared with G 01, shown in percentages. Values >100% compared to G 01 are highlighted in red. Values <100% compared to G 01 are highlighted in green. Max, maximum.

To investigate the stability of reduction guides during osteotomy surgery, the parameters listed in Table 1 were modified and analyzed (Figure 4). G 00 was chosen as a starting point guide. The influence of placing the guide flush on the bone versus offsetting it 10 mm was investigated in a first iteration. Then the influence of different widths and heights of the guide was studied. The influence of different cross-section profiles (T and H profiles) was investigated. The diameter of the K-wires was varied, and the position of the K-wire slots was changed. The guide construct was reinforced with longitudinally insertable K-wires, and in a second step, 2 longitudinal slots were added in the guide to insert 2 small osteotomes vertically to potentially stabilize the guide construct. Furthermore, the orientation of the K-wire slots was changed from longitudinal to transverse and its trajectory was varied.

**Table 1:** Overview of Different Guide Designs^*a*^

Guide Variants	Description	Dimensions (in Relation to G 00)
**G 00**	Basis design	l: 85 mm, w: 15 mm, h: 15mm, a: 36 mm, b: 14 mm, d: 4 mm
***0. Distance to bone***		
**G 01**	G 00 flush on the bone	
**G 02**	10-mm distance between bone and guide G 00	
***1. Height***		
**G 11**	+ 25% higher	h’ = 1.25 × h
**G 12**	− 25% lower	h’ = 0.75 × h
***2. Width***		
**G 21**	+ 25% wider	w’ = 1.25 × w
**G 22**	− 25% narrower	w’ = 0.75 × w
***3. Profile***		
**G 31**	T profile	e = 0.25 × h, e = 0.75 × w
**G 32**	H profile	e = 0.25 × h, f = 0.75 × w
**G 35**	T profile with same cross section as G 00	e = 0.25 × h, f = 0.25 × w, w’ = 1.75 × w, h’ = 1 × h
**G 36**	H profile with same cross section as G 00	e = 0.25×h, f = 0.25*w, w’ = 1.5 × w, h’ = 1 × h
***4. K-wire diameter***		
**G 41**	+ 25% thicker K-wire slot	d’ = 1.25 × d (5 mm)
**G 42**	− 25% thicker K-wire slot	d’ = 0.75 × d (3 mm)
***5. K-wire distance***		
**G 51**	+ 50% interfragmentary distance	a’ = 1.5 × a, b’ = 1 × b
**G 52**	− 50% interfragmentary distance	a’ = 0.5 × a, b’ = 1 × b
**G 55**	+ 25% intrafragmentary distance	a’ = 1 × a, b’ = 1.5 × b
**G 56**	− 25% intrafragmentary distance	a’ = 1 × a, b’ = 0.5 × b
***6. Reinforcement***		
**G 61**	1× longitudinally insertable K-wire	K-wire diameter = 2 mm
**G 62**	2× longitudinally insertable K-wire	K-wire diameter = 2 mm
**G 63**	4× longitudinally insertable K-wire	K-wire diameter = 2 mm
**G 64**	2× longitudinally insertable osteotomes	osteotome height = 2 mm, width = 8 mm
***7. Transverse K-wires***		
**G 71**	G 00 with slightly offset pins mirrored	a’ = 1 × a, b’ = 1 × b, offset from midline = 2.5 mm
**G 72**	G 00 with slightly offset pins inverted	a’ = 1 × a, b’ = 1 × b, offset from midline = 2.5 mm
**G 74**	G 21 with 2 transversely placed K-wire holes, interfragmentary wide	h’ = 1.25 × h, a’ = 1.78 × a
**G 75**	G 21 with 2 transversely placed K-wire holes, interfragmentary narrow	h’ = 1.25 × h, a’ = 1 × a
***8. Diverging K-wires***		
**G 81**	G 11 with K-wire holes placed 15° diverging	
**G 82**	G 75 with K-wire holes placed 15° diverging	

a *a*, interfragmentary K-wire distance; *b*, intrafragmentary K-wire distance; *d*, K-wire slot diameter; *e*, beam height; *f*, beam width; *h*, height; *l*, length; *w*, width; H profile, profile with the cross section shape of a H turned by 90°; T profile, profile with the cross section shape of a T.

### FEA and Material Properties

For FEA, Ansys Workbench (Version R1; Ansys Inc) was used to create a mesh using tetragonal elements with an approximate element size of 0.5 mm for the guide and the K-wires. A convergence analysis was performed and an aspect ratio of <3 was recorded for 95% of its elements. For the reduction guide, biocompatible polyamid P2200 is commonly used as the material for 3D laser sintering process^25,27^ and was modeled as linear isotropic material property (*E* = 1.65 GPa; *v* = 0.35).^7^ Stainless steel material properties were assigned to the K-wires and osteotomes (*E* = 193 GPa; *v* = 0.31).

### Loading and Boundary Conditions

**Figure 5.**
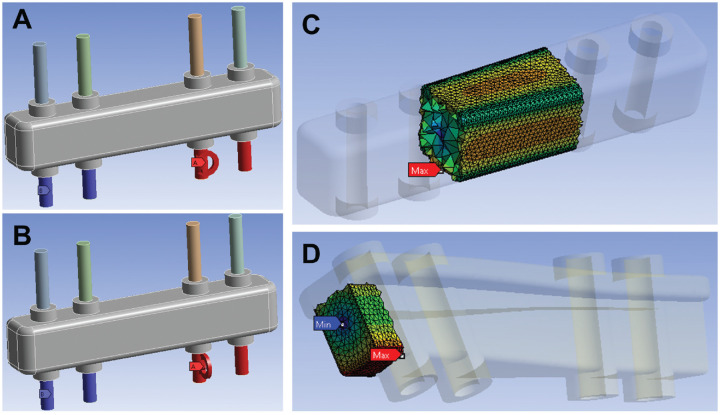
Boundary conditions for test setup. The interior area of the blue K-wires was fully constrained, whereas at the interior area of the remaining K-wires a moment of 50 Nm was applied in (A) longitudinal (bending) and (B) transverse (torsion) directions. (C) The equivalent stress (mean and maximum) for the guide was recorded in a segment between the 2 K-wires, whereas (D) the maximum deformation of the guide was anaylzed from a defined box at the end of the guide that has the same exact position and size for all different guides to account for the different designs. Max, maximum; Min, minimum.

Two K-wires were considered fully constrained inferior to the guide. On the remaining 2 K-wires, an arbitrary moment of 50 Nm was applied in the longitudinal direction of the guide, hereinafter termed “bending.” In a second load step, a 50-Nm moment in the transverse direction of the guide was applied to the same K-wires, hereinafter termed “torsion” (Figure 5). These 2 loading conditions correspond to the main loading of a reduction guide intraoperatively (bending for open or closing wedge osteotomies and torsion for rotational osteotomies).^3,13^ The contact between the K-wires and their corresponding slots in the reduction guide were modeled as fully bonded in Ansys Workbench, allowing no separation or sliding between the contact surfaces to support a linear solution.^14^ For the reinforced guides, the contact between the guide and the K-wires respectively, the guide and the osteotomes was modeled as fully bonded as well.

### Outcome Measures

For the reduction guide, an area of interest to analyze equivalent stress was defined in the central region of the guide between the 2 centrally placed K-wire slots to reduce a possible influence of different K-wire hole spacing and contact conditions on the local stress environment of the guide (Figure 5C).^14^ The length of the segment was identical for all guide designs. Mean equivalent stress was read out for this segment. To determine the maximum deformation of the guide during loading, an area of interest was defined at the loaded end of the guide (Figure 5D). This box had the identical dimensions and absolute position for each guide configuration to allow a guide deformation measurement independent of the different guide cross sections. An additional advantage of this method was that the deformation of the entire guide construct (guide + K-wires) was mapped through this box, as the K-wires were considered via contact connections. The maximum deformation and equivalent stress of the K-wires was read out separately for each load case.

## Results

All results are reported relative to the guide design G 01 (basic guide design G 00 placed flush on bone surface) and presented in detail in Figure 4 and Table 2. Placing the guide with an offset of 10 mm to the bone surface compared with flush on the bone, a 4% increase in guide and K-wire deformation was recorded for bending. For torsion, a 61% increase of guide deformation and 17% increase of K-wire deformation were measured (Table 2). A 25% width increase led to an 18% reduction of guide deformation for bending and 22% for torsion.

**Table 2:** Overview of Tested Guide Designs for Bending and Torsion Load Cases^*a*^

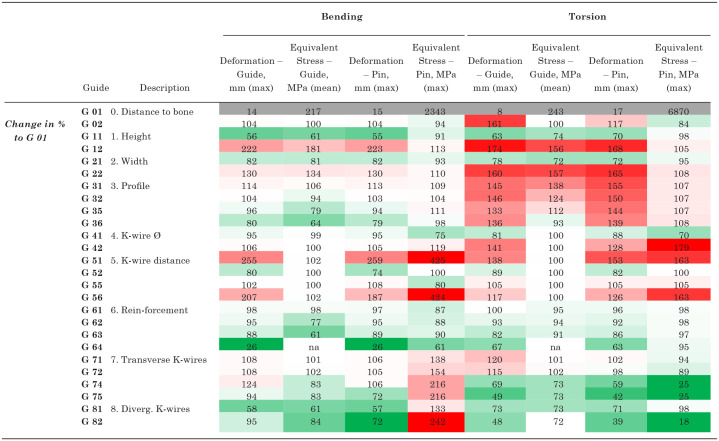

a Results are presented in % relative to G 01. Values >100% compared to G 01 are highlighted in red. Values < 100% compared to G 01 are highlighted in green. Diverg., diverging; max, maximum; na, not applicable.

A 25% height increase led to a 44% reduction of guide deformation for bending. This corresponded to the best guide design for bending, neglecting reinforcement configurations. The best performance for bending was shown by G 64, where two 8-mm osteotomes were inserted longitudinally for reinforcement (74% guide deformation reduction). The best design for torsion was G 82, where the K-wire slots were placed transversely and the trajectory of the K-wire slots was 15° diverging (52% guide deformation reduction). Increasing the K-wire diameter from 4 mm to 5 mm led to a 5% guide deformity reduction in bending and 19% in torsion, respectively.

## Discussion

The main findings of this study were that the guide height was the most important factor influencing the guide stability for bending. The placement of the K-wire slots transverse to the guide axis influenced the guide stability most positively for the torsional load case.

Placing the reduction guide flush on the bone surface reduced guide and K-wire deformation and is a measure that can be easily implemented intraoperatively without additional effort.

Increasing the guide height had a greater effect than guide widening on bending stability, as the moment of inertia of the cross section in the direction of loading was increased.^2^ The fact that a higher guide performed better in torsion compared with a wider guide, even though their cross sections had the same polar moment of inertia, can be explained with the increased K-wire sleeve stability due to the increased height of the guide.^19^ Increasing the guide height had the added benefit that, unlike widening, it did not require additional intraoperative exposure of the bone.

Modifying the cross section to an H or T profile with the same outer dimensions as the basic guide (G 00) barely affects bending stability but clearly increases torsional deformation by 46% due to the reduced moment of inertia in the *x*-direction. H and T profiles are designed to absorb high bending moments combined with weight optimization in civil engineering. Since the weight of a guide is not relevant for the orthopaedic application, such profiles do not offer any benefit.

The effect of increasing the K-wire diameter is more relevant for torsion then for bending in the tested configurations and is a simple way to improve construct stability due to the fact that the bending stiffness of a metallic rod changes as the fourth power of the diameter.^1^ Therefore, increasing the diameter of the K-wires by 50% will increase the stiffness by a factor of 5. Increasing the diameter of the K-wire infinitely does not appear to be a viable solution. This is because infinite stability of the metallic K-wire puts more load on the polyamid guide, which is already weaker, resulting in greater deformation of the entire construct. In all tested configurations, it was observed that there is a correlation between improved guide stability and reduced guide stress, K-wire deformity, and maximum K-wire stress.

It is crucial to position the first K-wire slots as close as possible to the osteotomy site by reducing the interfragmentary K-wire distance to minimize guide deformation. Increasing the interfragmentary K-wire slot distance and decreasing the intrafragmentary distance, however, increases guide deformation and leads to a potential plastic deformation of the K-wires due to severe stress and deformation increase. Therefore, it is most effective to place the central K-wire slot as close as possible to the osteotomy site and spread out the intrafragmentary distance of the slots.

If maximum guide stability is necessary, reinforcing the polyamid guides with commercially available insertable K-wires or osteotomes is an option. Inserting 2 osteotomes provided superior stability compared with K-wires and resulted in the least guide deformation of all tested configurations for bending (74% less guide deformation), due to the high moment of inertia in the *y*-direction of the vertically inserted osteotomes.^19^ Guides with slots for osteotome reinforcement could be easily printed and assembled intraoperatively for open or closing wedge osteotomies with high bending moments.

For femoral or tibial rotational osteotomies for patellofemoral instability,^13^ where torsion might be the main loading condition for the reduction guide, placing the 2 K-wire slots transversely to the guide direction and parallel to the osteotomy line significantly increases torsional stability of the guide. It is important to position the K-wire slots as close as possible to the osteotomy line to achieve the highest torsional stability. Additionally, diverging the trajectories of the K-wire slots does not relevantly improve bending or torsional stability for any tested configurations.

### Limitations

Limitations of this study include the use of a simplified reduction guide based on the dimensions of a commonly used distal femoral reduction guide for closing or opening wedge distal femoral osteotomies. The 2 most expected load cases were simulated. However, depending on the osteotomy location and the effective deforming forces, the perceived intraoperative direction of deformation may vary, especially in osteotomies where no hinge was left or in cases where the hinge broke accidentally during the procedure. Due to the printing procedure of selective laser sintering, the material properties slightly varies depending on their orientation in the 3D printer.^6,17^ Therefore, anisotropic, heterogeneous guide material was modeled using isotropic and homogeneous material properties and a linearly elastic analysis was performed. This is a common method and does not discredit the differences found between the different guide designs.^20^ It is important to highlight that as this model assumed linear elasticity, it is not possible to simulate fracture or plastic deformation. Similar approaches using linear elasticity have been previously shown to work well.^9^ Modeling the guide in combination with the K-wires allowed prediction of the deformation of the entire construct; however, this introduced additional complexity into the model, since contact conditions had to be moderated. Assuming fully bonded contact conditions between the K-wire and the reduction guide is reasonable with the rationale that there are negligible motions at these interfaces during subfailure conditions.^14^ However, this assumption did affect the local stress environment, and to account for that, equivalent stress was only analyzed in a central region of the guide with enough distance from the K-wire slots.

## Conclusion

The PSI reduction guides should be placed flush on the bone, and K-wires with a diameter of ≥4 mm should be used. The most effective methods to increase reduction guide stability for all load cases are to increase the guide height and reduce the central K-wire distance to the osteotomy. When performing opening or closing wedge osteotomies, which mainly involve bending of the PSI guide, a high-profile guide and longitudinally oriented K-wire slots should be used. On the other hand, when torque is expected, as in rotational osteotomies, the K-wire holes in PSI guides should be oriented transversely to reduce intraoperative deformation.
